# A high-speed gear reshaping method for electric vehicles combining the effects of input torque and speed variation

**DOI:** 10.1371/journal.pone.0302814

**Published:** 2024-06-10

**Authors:** Weifeng Liu, Cuicui Wei, Bo Wang, Zhicheng Ding, Guitao Du

**Affiliations:** School of Mechanical and Electrical Engineering, Zhengzhou University of Industrial Technology, Zhengzhou, China; Ghani Khan Choudhury Institute of Engineering and Technology, INDIA

## Abstract

In this study, we introduce an optimization method for high-speed gear trimming in electric vehicles, focusing on variations in input torque and speed. This approach is designed to aid in vibration suppression in electric vehicle gears. We initially use Tooth Contact Analysis (TCA) and Loaded Tooth Contact Analysis (LTCA) to investigate meshing point localization, considering changes in gear tooth surface and deformations due to load. Based on impact mechanics theory, we then derive a formula for the maximum impact force. A 12-degree-of-freedom bending-torsion-axis coupled dynamic model for the helical gear drive in the gearbox’s input stage is developed using the centralized mass method, allowing for an extensive examination of high-speed gear vibration characteristics. Through a genetic algorithm, we optimize the tooth profile and tooth flank parabolic modification coefficients, resulting in optimal vibration-suppressing tooth surfaces. Experimental results under various input torques and speeds demonstrate that the overall vibration amplitude is stable and lower than that of conventional gear shaping methods. Specifically, the root mean square of vibration acceleration along the meshing line under different conditions is 58.02 m/s^2^ and 20.33 m/s^2^, respectively. The vibration acceleration in the direction of the meshing line is 20.33 m/s^2^ and 20.02 m/s^2^ under varying torques and speeds, with 20.33 m/s^2^ being the lowest. Furthermore, the average magnitude of the meshing impact force is significantly reduced to 5015.2. This high-speed gear reshaping method not only enhances gear dynamics and reliability by considering changes in input torque and speed but also effectively reduces vibration in electric vehicle gear systems. The study provides valuable insights and methodologies for the design and optimization of electric vehicle gears, focusing on comprehensive improvement in dynamic performance.

## 1. Introduction

A gear train is a mechanical system with inherent elasticity that, when driven by dynamic excitation, produces significant vibration and noise. This dynamic excitation can be primarily categorized into internal and external excitations. During the meshing process of a gear pair, internal excitation arises due to factors such as changes in the number of teeth engaged, elastic deformation of gear teeth under load, errors in gear manufacturing and installation, and friction between tooth surfaces. This internal excitation primarily encompasses time-varying mesh stiffness, mesh impact, and tooth surface friction excitations. Apart from the internal excitation during gear pair meshing, dynamic excitation attributed to other factors is referred to as external excitation. External excitations mainly arise from factors including geometric eccentricity of the gear pair, torque fluctuations between the prime mover and the load, time-varying stiffness of the rolling bearings, and clutch nonlinearity.

Gears constitute a crucial component of slewing gearboxes, and the presence of fine gears complicates the effective identification of response meshing, underscoring the importance of enhancing computational accuracy in dynamic simulations, especially in the context of microcracking. Ma et al. [1] conducted simulations to calculate signals for teeth of varying depths, considering different tooth parameters. The impact of cracks was assessed using fault detection indices, root mean square (RMS) values, kurtosis, and crest factors. Zheng et al. [2] introduced a quadruple linkage shaping method, which employs the coordinated movement of four cutter axes to shape gears. This approach begins with the establishment of a mathematical model, followed by an analysis of the cutting process, and culminates in experiments demonstrating the method’s effectiveness. The performance of electric vehicles is directly linked to the accuracy of gear shaping, with improvements in gear reliability contributing to reduced noise and vibration during high-speed operations [3]. To further mitigate gear friction vibration, Jiang et al. [4] developed a novel two-part gear friction dynamics model that incorporates tooth profile modification (TPM). The findings indicate that excessive and minimal power indices intensify vibration contact pressure, and notably, uncertainty significantly affects fatigue life. Gear shaping has been identified as an effective technique to address this issue. Mesh contact analysis, taking into account corrections and assembly error processing, yields accurate results under simulated real-world conditions. Consequently, Cheng et al. [5] proposed a multi-objective optimization design method aimed at reducing spiral vibration and noise.

The proposed method is illustrated with examples. The results demonstrate that the optimized root mean square (RMS) value is reduced by nearly fourfold, achieving a surface difference improvement of approximately 320 times, indicating high effectiveness. Gear reshaping has been proven to significantly enhance the dynamic characteristics of the transmission system and markedly reduce the likelihood of vibration deterioration, wear deviation, and fatigue fracture. Gao et al. [6] introduced an optimization technique for involute straight-toothed cylindrical gears based on a transverse torsion-swing coupled nonlinear model system. This technique involves selecting tooth shape parameters as independent variables and utilizing target load coefficients and driving wheel speed torque to establish boundary conditions. The analysis compares responses across the time domain, frequency phase plane, Poncalais cross-section, and considers the maximum Lyapunov exponent. Liu et al. [7] demonstrated that micromodified shape optimization, using the gear system simulation software MASTA, can effectively mitigate whistling noise. The establishment of the calibration model was achieved through contact point testing and calibration. A study on micro-trimming shape was conducted to reduce gear transmission error and optimize tooth shape. Min et al. [8] achieved quality improvement by overcoming forging constraints and established an accurate envelope motion model between tool and workpieces. This model, based on velocity mapping relationships, facilitates the shaping process for each machine axis, enabling the finalization of complex variable-toothed racks and the derivation of specific contour mathematics through an associative solution model, ultimately allowing for predictive modeling. Zhou et al. [9] focused on designing top-trimmed toothed non-circular gears that conform to a specific complex ratio curve. They proposed a criterion for the bottom cut of profile distortion and derived a theoretical model based on the generating principle of the shaping tool, employing a series of discrete point-set pitch curves. Pillarz et al. [10] investigated the use of laser triangulation and confocal color gear shape measurement through a transverse scanning position measurement method, effectively addressing the issue of limited measurement accuracy in gear shaping.

**Fig 1:**
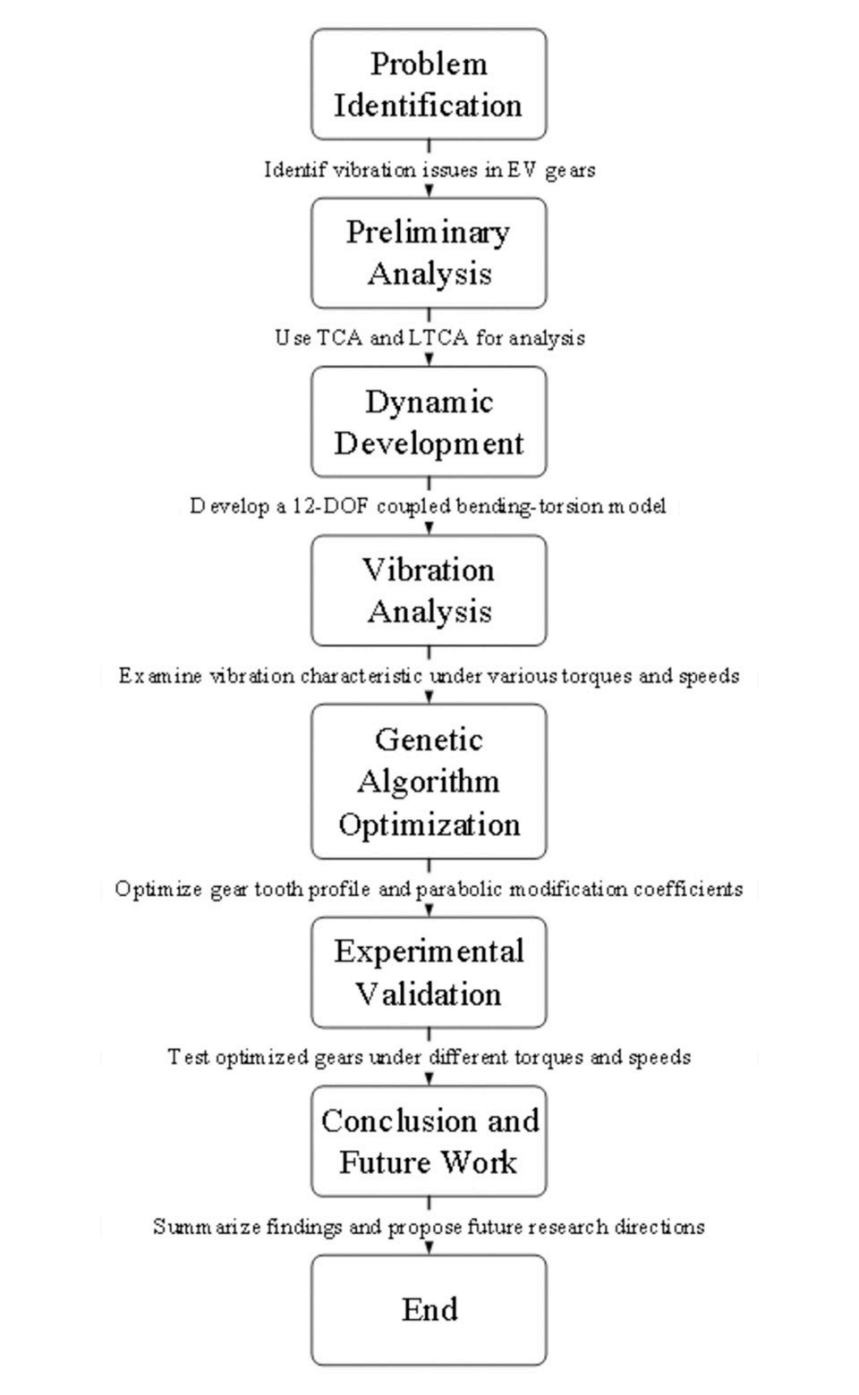
Flowchart of the proposed work.

Despite extensive research on gear noise and vibration, the primary focus has been on error measurement and accuracy, with less consideration given to the influence of multiple factors in gear shaping. In response to this gap, this paper introduces a gear shaping optimization method that incorporates changes in input torque and speed, aiming to serve as a reference for suppressing vibration in electric vehicle gears. The proposed workflow is depicted in Fig 1.

The novel contributions of this study are manifested in several key areas:

1. Holistic Approach to Input Torque and Speed Variations: The study introduces an innovative gear reshaping strategy characterized by its comprehensive consideration of input torque and speed variations. Contrary to previous research, which generally focuses on optimizing gear performance under singular operating conditions, this approach integrates the impacts of both input torque and speed variations.
2. Dynamic Performance Optimization Across Multiple Cases: The proposed strategy excels beyond conventional methods by improving gear dynamic performance under a range of operating conditions, especially in vibration and noise control. This results in a comprehensive enhancement of performance, representing a significant advancement from methods focusing on limited operational scenarios.
3. Addressing Vibration Issues Stemming from Dynamic Excitation and Elastic Deformation: The proposed method effectively addresses vibration issues arising from dynamic excitation and elastic deformation in gears. This advancement enhances the reliability and efficiency of gears under diverse conditions and is critically important for improving the overall performance and driving comfort of electric vehicles. By tackling these challenges, our approach significantly contributes to the field, opening new avenues for the optimization of electric vehicle gear systems.

## 2. Engagement impact force calculation

### 2.1 Determination of engagement points

**Fig 2:**
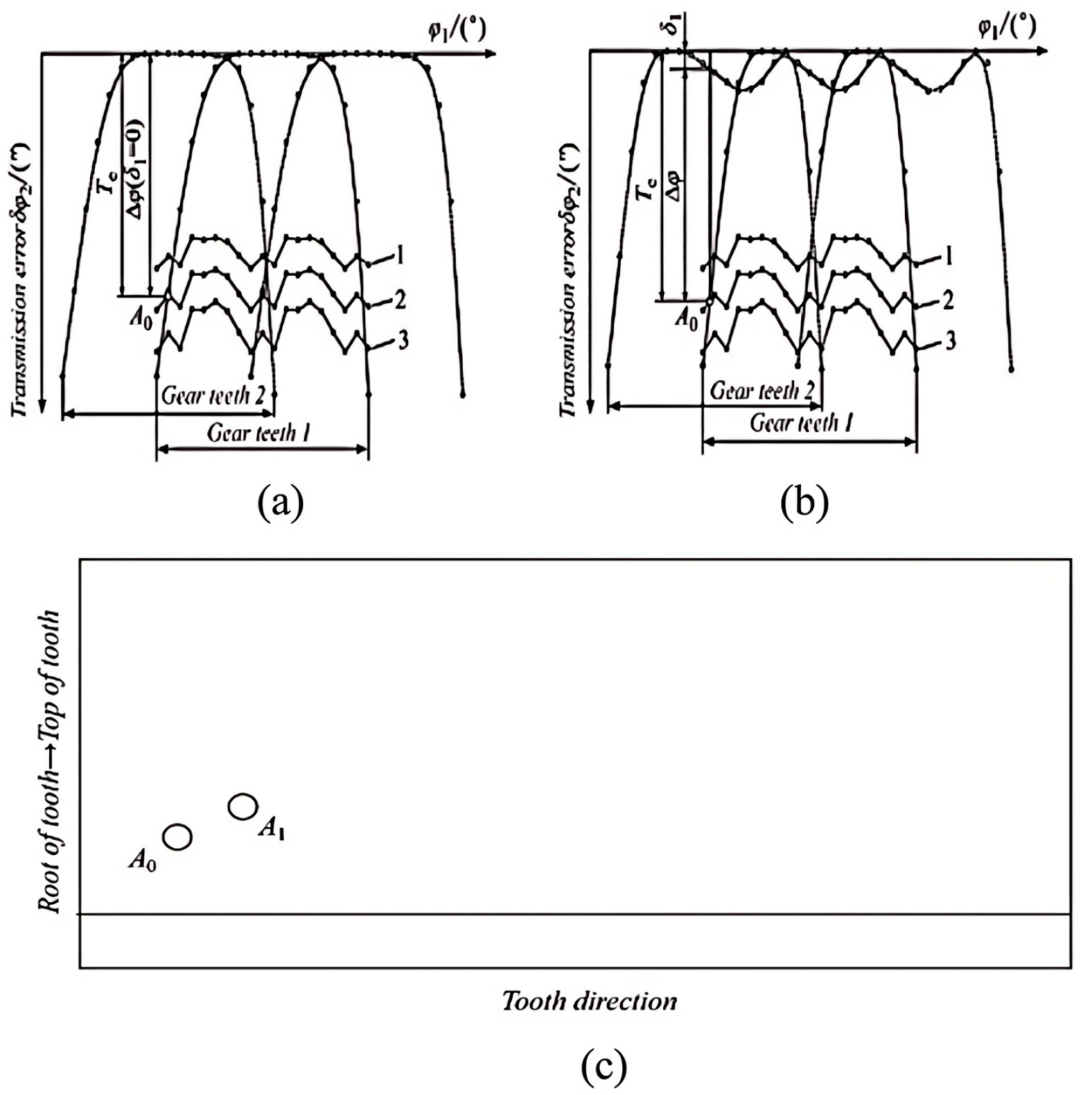
The solution of the actual meshing point of the tooth surface. (a) the solution of the actual meshing point of the conventional medium-convex modified tooth surface; (b) the solution of the actual meshing point of the newly modified tooth surface; (c) the schematic diagram of the actual meshing point of the tooth surface. Note: 1 is LTE for load 1; 2 is LTE for load 2; 3 is LTE for load 3.

The key to calculating the meshing impact force lies in the precise localization of the meshing point’s position. After gear reshaping, the contact zone is retained on only a portion of the tooth surface. Fig 2 presents a schematic diagram of a reduction gear, illustrating that the actual engagement point does not commence at the apex of the large gear teeth, but rather at a specific point on the tooth surface. This paper investigates a solution for localizing the meshing entry point, considering the effects of surface variations and load deformation of the gear teeth. During the load transfer between a pair of gears, as depicted in Fig 2(A) and 2(B), the pinion contracts and the larger gear expands at the corresponding base, resulting in the premature union of teeth. At this juncture, the starting position of the large tooth’s meshing can be considered to have retreated by a slight angle from the theoretical position. This angle can be determined using the LTCA technique, based on the instantaneous contact point of the meshing small tooth surface.

According to Fig 1(A) and 1(B), TE curves and LTE curves can be derived by applying TCA and LTCA techniques. In Fig 2(A) and 2(B), the TE corresponding to wheel tooth 2 at the theoretical engagement position is *δ*_*1*_, *T*_*e*_ is the load transmission error, and Δ*φ* represents the small angle at which the large wheel tooth that starts to engage is retracted from the theoretical position. *A*_*0*_ in Fig 1(C) is the theoretical engagement position, while *A*_*1*_ is the actual engagement position.


$$\Delta \varphi =T_{e}-\delta_{1}$$ (1)

### 2.2 Calculation of impact force

After determining the position of the meshing point, the relative normal velocities of the two tooth surfaces and the meshing stiffness can be calculated from the TCA and LTCA. The moment of inertia of the meshing gear pair is.


$$\left\{ \begin{array}{l} J_{1}=\frac{\pi \rho b}{2}\left(r_{b1}^{4}-r_{h1}^{4}\right)\\ J_{2}=\frac{\pi \rho b}{2}\left(r_{b2}^{4}-r_{h2}^{4}\right) \end{array}\right.$$ (2)

The conversion of the moment of inertia of two meshing gears into an induced mass on the instantaneous meshing line is done with the help of the formulas for the conversion of moment of inertia and mass in mechanics. According to these formulas, the moment of inertia of the gears can be converted into an induced mass on the instantaneous meshing line in order to better analyze the dynamic performance of the gear system.


$$\left\{ \begin{array}{l} m_{red1}=\frac{J_{1}}{r_{b1}^{2}}\\ m_{red2}=\frac{J_{2}}{r_{b2}^{2}} \end{array}\right.$$ (3)

Eq (2) and (3): *J*_*1*_ is the instantaneous moment of inertia of the small wheel; *J*_*2*_ is the instantaneous moment of inertia of the large wheel; *b* is the width of the tooth; *ρ* is the density of the gear material; *r*_*b1*_ is the radius of the base circle of the small wheel; *r*_*b2*_ is the radius of the base circle of the large wheel; *r*_*b1*_ and *r*_*b2*_ are the radius of the inner hole of the hub of two gears, respectively; *m*_*red1*_ and *m*_*red2*_ are the induced mass of the instantaneous meshing line.

The kinetic energy of the impact of the gear pair meshing into the point of impact *E*_*k*_ is.

$$E_{k}=1/2\times \frac{J_{1}J_{2}}{\left(J_{1}r_{b2}^{2}+J_{2}r_{b1}^{2}\right)}\times v_{s}^{2}=\int_{0}^{\delta_{S}}K_{S}x$$ (4)

Where, *v*_*s*_ is the relative normal velocity between the small wheel tooth surface and the large wheel tooth surface at the initial contact point.

According to the theory of impact mechanics, the maximum impact force *F*_*s*_ can be derived from the impact kinetic energy. Due to the impact effect, the impact deformation *δ*_*s*_ will be generated between the gear teeth, and the corresponding impact force *F*_*s*_ is the maximum impact force:

$$F_{s}=K_{S}\delta_{S}=v_{S}\sqrt{\frac{J_{1}J_{2}}{\left(J_{1}r_{b2}^{2}+J_{2}r_{b1}^{2}\right)}K_{S}}$$ (5)

where, *K*_*s*_ is the meshing stiffness between the small wheel tooth surface and the large wheel tooth surface at the initial contact point.

## 3. High-speed gear transmission vibration analysis

### 3.1 High-speed gear system dynamics equation construction

The focus of this study is a pure electric vehicle’s single-stage, two-stage helical gear reducer, aiming to comprehensively explore the vibration characteristics of high-speed gears. Employing the concentrated mass method, this study constructed a 12-degree-of-freedom dynamic model that integrates bending, twisting, and axis coupling for the helical gear transmission at the reducer’s input stage [11]. Utilizing Newton’s second law and the decomposition of forces and displacements [12], the dynamic meshing force of the high-speed gear drive, expressed in parametric form along the x, y, and z axes, can be derived as follows:

$$\left\{ \begin{array}{l} F=k_{m}\left[\begin{array}{l} \left(x_{1}-x_{2}\right)\sin\phi_{1}\cos\beta +\left(y_{1}-y_{2}+r_{b1}\theta_{1z}-r_{b2}\theta_{2z}\right)\cos\phi_{1}\cos\beta \\ +\left(z_{1}-z_{2}+r_{b1}\theta_{1x}-r_{b2}\theta_{2x}\right)\sin\phi_{1}+\left(r_{b1}\theta_{1y}-r_{b2}\theta_{2y}\right)\cos\phi_{1}\sin\beta \end{array}\right]\\ \;+c_{m}\left[\begin{array}{l} \left({\dot{x}}_{1}-{\dot{x}}_{2}\right)\sin\phi_{1}\cos\beta +\left({\dot{y}}_{1}-{\dot{y}}_{2}+r_{b1}{\dot{\theta }}_{1z}-r_{b2}{\dot{\theta }}_{2z}\right)\cos\phi_{1}\cos\beta \\ +\left({\dot{z}}_{1}-{\dot{z}}_{2}+r_{b1}{\dot{\theta }}_{1x}-r_{b2}{\dot{\theta }}_{2x}\right)\sin\phi_{1}+\left(r_{b1}{\dot{\theta }}_{1y}-r_{b2}{\dot{\theta }}_{2y}\right)\cos\phi_{1}\sin\beta \end{array}\right]\\ F_{x}=F\sin\phi_{1}\cos\beta \\ F_{y}=F\cos\phi_{1}\cos\beta \\ F_{z}=F\sin\beta \end{array}\right.$$ (6)

where *x*_*1*_, *x*_*2*_ are the vibration displacements of the 1st and 2nd gears along the x-axis, respectively; *y*_*1*_, *y*_*2*_ are the vibration displacements of the 1st and 2nd gears along the y-axis, respectively; *z*_*1*_, *z*_*2*_ are the vibration displacements of the 1st and 2nd gears along the z-axis, respectively; *θ*_*1x*_, *θ*_*2x*_ are the oscillatory displacements of the 1st and 2nd teeth around the x-axis, respectively; *θ*_*1y*_, *θ*_*2y*_ are the oscillatory displacements of the 1st and 2nd The first and second teeth are the oscillating displacement around the y-axis; *θ*_*1z*_ and *θ*_*2z*_ are the torsional displacement around the z-axis; *F*_*x*_ is the dynamic meshing force along the x-axis; *F*_*y*_ is the dynamic meshing force along the y-axis; *F*_*z*_ is the dynamic meshing force along the z-axis; *ϕ*_1_ is the angle between the tooth-pair meshing plane and the positive direction of the y-axis; *F* is the dynamic meshing force in the direction of the meshing line; *k*_*m*_ is the time-varying meshing stiffness of the gears; *c*_*m*_ is the time-varying meshing damping of the gears. gear time-varying mesh damping; *β* is helix angle of helical gear.

The engagement damping *c*_*m*_ is given by:

$$c_{m}=2\xi \sqrt{\frac{k_{m}J_{1}J_{2}}{J_{1}r_{b2}+J_{2}r_{b1}}}$$ (7)

where, *ξ* is the damping ratio, which is set to 0.1 in this study with reference to related scholars [13, 14].

Considering that the gear rigid body angular displacement may lead to some deviation of the results in the analysis, in order to eliminate this error, this study adopts the line displacement instead of angular displacement, The specific expression for its displacement *q* is given by:

$$q=r_{b1}\theta_{1z}-r_{b2}\theta_{2z}$$ (8)

According to Eq (8), the expression for the torsional vibration equation can be simplified:

$$\left\{ \begin{array}{l} \overset{¨}{q}m_{e}+F_{y}+F_{st}+F_{f}\frac{S_{1}}{r_{b1}}=\frac{T_{1}}{r_{b1}}\\ m_{e}=\frac{I_{1}I_{2}}{I_{1}r_{b2}^{2}+I_{2}r_{b1}^{2}} \end{array}\right.$$ (9)

Where, *m*_*e*_ is the equivalent torque mass of the gear pair; *F*_*st*_ is the meshing impact force; *F*_*f*_ is the tooth surface friction; *S*_*1*_ is the friction arm of each wheel; and *T*_*1*_ is the input torque of the high-speed class helical gear system.

Given the system’s simplicity, this study employs the Runge-Kutta method [15] to solve the dynamic equations of the gear system. To reduce computational complexity, this study synthesizes gear vibration accelerations across various directions, yielding the relative vibration acceleration along the end face meshing line direction. This serves as the primary index for conducting vibration evaluation of the gear transmission system.


$$\begin{array}{l} a=\left[\left({\overset{¨}{x}}_{1}-{\overset{¨}{x}}_{2}\right)\sin\phi +\left({\overset{¨}{y}}_{1}-{\overset{¨}{y}}_{2}+\overset{¨}{q}\right)\cos\phi \right]\cos\beta \\ \;+\left[\left({\overset{¨}{z}}_{1}-{\overset{¨}{z}}_{2}+r_{b1}{\overset{¨}{\theta }}_{1x}-r_{b2}{\overset{¨}{\theta }}_{2x}\right)\sin\phi_{1}+\left(r_{b1}{\overset{¨}{\theta }}_{1y}-r_{b2}{\overset{¨}{\theta }}_{2y}\right)\cos\phi \right]\sin\beta \end{array}$$ (10)

### 3.2 Vibration analysis of high-speed gearing

**Fig 3:**
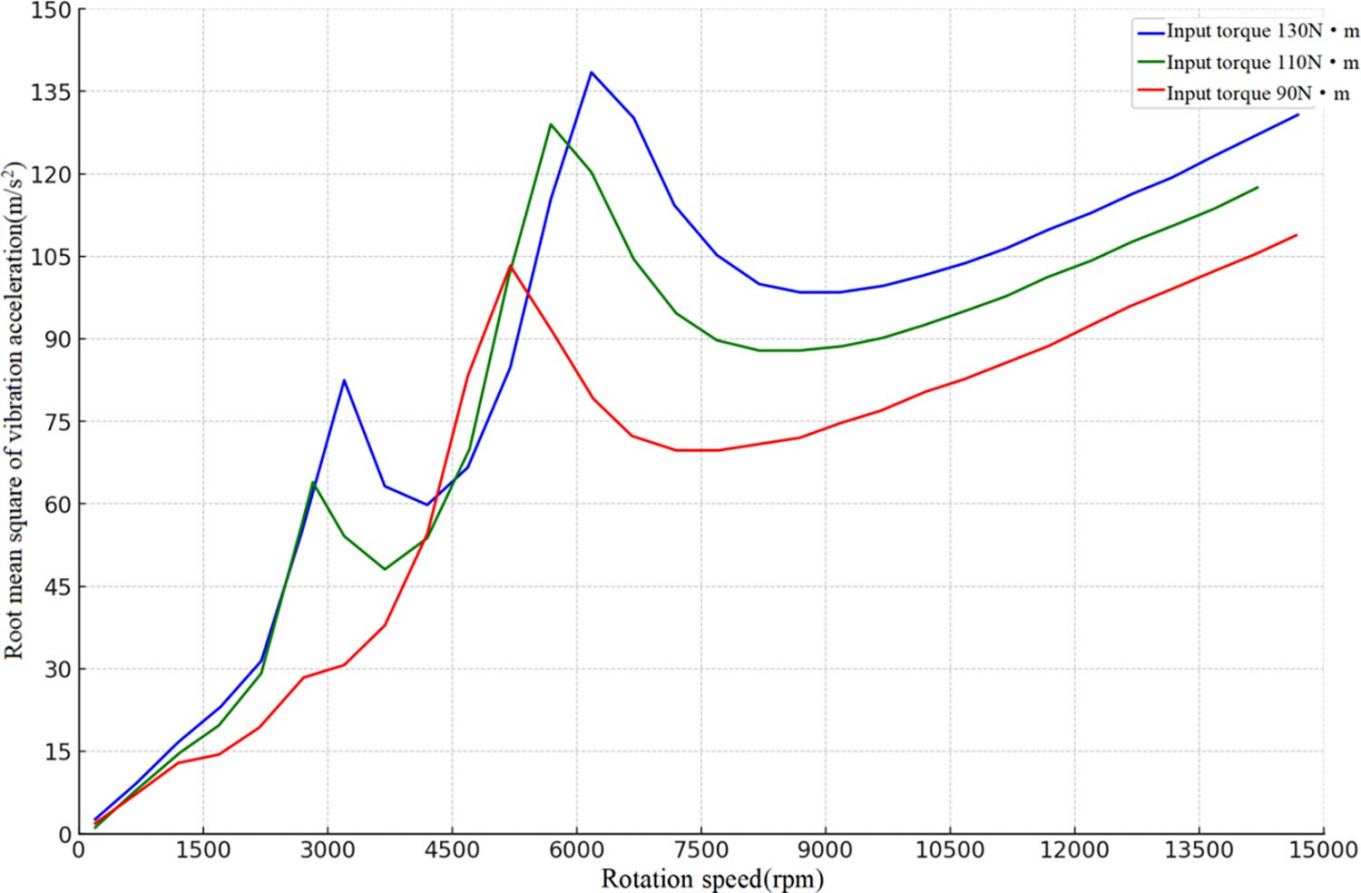
Variation of vibration speed in the direction of meshing line under combined excitation.

The vibration characteristics of the high-speed stage helical gear train exhibit distinct features at input torques of 90N-m, 110N-m, and 130N-m, respectively. Under the influence of combined excitation, Fig 3 depicts the vibration variations along the meshing line direction at different rotational speeds. At rotational speeds of 1/3 and 1/2 of the resonant speeds, the system exhibits a superharmonic resonance phenomenon in both instances. Except in the peak resonance region, there is a consistent increase in the rms of vibration acceleration along the engagement line direction with input speed, with both parameters amplifying in the same direction. The primary reason for this phenomenon is that stiffness excitation amplitude remains constant despite changes in rotational speed. However, a continuous increase in input rotational speed positively influences the integrated excitation, impacting the meshing force and causing continuous variations, thereby increasing the vibration amplitude during the meshing impact process. Consequently, an acceleration in input speed leads to a continuous enhancement of system vibration within the over-resonance region, albeit this enhancement is markedly less pronounced than in scenarios involving solely shock excitation. Additionally, combined excitation encompasses time-varying meshing stiffness excitation, thus increasing input torque results in an accelerated system resonant speed.

In high-speed gear systems with varying input torques, the acceleration of vibration along the meshing line direction is subject to continuous change in response to increases in input torque and speed, manifesting a pronounced periodicity within the time domain response. The presence of meshing shock excitation contributes to periodic sharp transitions in the directional vibration acceleration of the meshing line, with its significant variations primarily dictated by the input torque and speed. The system’s frequency domain response reveals that the amplitude of vibration escalates with the input torque increment. Even at 20 times the frequency, owing to meshing shock excitation, the amplitude of vibration is discernible. At input speeds of 8000rpm and 13000rpm, the frequency domain amplitude peaks at 1x the tooth frequency, whereas at an input speed of 3000rpm, the maximal amplitude is observed at 2x the tooth frequency, heavily reliant on the resonance speed magnitude. Quantitative analysis indicates discernible peak values in vibration data at input torques of 130Nm and 110Nm. At 130Nm, the observed mean vibration acceleration is 88.18 m/s^2^, with a standard deviation of 38.87 m/s^2^ and a peak of 138.44 m/s^2^. For 110Nm torque, the corresponding values are a mean of 78.45 m/s^2^, a standard deviation of 35.61 m/s^2^, and a peak of 128.98 m/s^2^. However, at 90Nm input torque, although the mean vibration acceleration decreases to 67.05 m/s^2^, the vibration data exhibits a steady ascending trend without a distinct peak. This indicates that with reduced torque, the system’s overall vibrational performance tends towards stability, and no abrupt peaks are observed at 90Nm. The absence of a pronounced peak in vibration acceleration at 90Nm torque may be attributed to the dynamic characteristics of the system. Specifically, this torque level may not reach the conditions necessary to induce system resonance, thereby not producing a peak. Furthermore, the optimization of the gear tooth surface is intended to diminish vibrations across the full torque range, which could result in the absence of significant peaks at certain torque levels, presenting a smoother acceleration curve instead. Therefore, despite the lack of observed significant peaks in vibration acceleration at 90Nm input torque, this outcome aligns with the objective of reducing vibrations across the entire operational range through gear tooth profile and parabolic modification coefficients optimized by a genetic algorithm.

## 4. High-speed gear shaping for electric vehicles combining the effects of input torque and speed changes

### 4.1 Tooth face trimming

In this paper, the tooth profile is reshaped from the perspective of gear tooth contact analysis, and the standard tooth profile is transformed into a parabolic tooth profile passing through the coordinate origin. Through the coordinate transformation to get the equation of the pinion face, the helical gear tooth profile direction is realized. The coordinate system *S*_*a*_ is established on the normal tooth profile of the tool and moves with the tool; the coordinate system *S*_*b*_ is established on the normal tooth surface of the tool and moves with the tool; the coordinate system *S*_*c*_ is established on the transverse tooth surface at the midpoint of the tooth width and tooth pitch; the coordinate system *S*_*d*_ is the subsequent coordinate system established by the pinion; and the coordinate system *S*_*e*_ is the fixed coordinate system established by the pinion. *d*_*0*_ is the normal tooth thickness at the half pitch circle, and *α*_*N*_ is the normal pressure angle. In the coordinate system *S*_*a*_, the coordinates of the normal tooth profile of the rack tool are as follows:

$$r_{a}(x_{a})={\left[\begin{array}{cccc} x_{a} & ax_{a}^{2} & 0 & 1 \end{array}\right]}^{T}$$ (11)

where, *a* is the tooth profile parabolic modification factor.

The constructed pinion tooth equations are as follows:

$$r_{d}(x_{a},d_{1},\varphi_{3})=M_{de}M_{ec}M_{cb}M_{ba}r_{a}(x_{a})$$ (12)

where, *φ* is the turning angle of the pinion during gear processing; *d*_*1*_ is the distance between the coordinate system *S*_*b*_ and the coordinate system *S*_*c*_ along the direction of *o*_*b*_*z*_*b*_ axis; *M*_*de*_, *M*_*ec*_, *M*_*cb*_, *M*_*ba*_ are the coordinate transformation matrices.

The basic law of tooth profile meshing states that when the tooth profiles of two gears are in contact at any position, the instantaneous ratio of the two gears to the common normal of the instantaneous point of contact must intersect the line of consecutive centers at a certain point *p*. Therefore, the rack and pinion tool must necessarily satisfy the meshing equation after contacting the pinion tooth surface:

$$f(x_{a},d_{1},\varphi_{3})={\vec{n}}_{c}\cdot {\vec{v}}_{c}^{\left(c,d\right)}=0$$ (13)

$$M_{ba}=[\begin{array}{cccc} -\cos\alpha_{n} & \sin\alpha_{n} & 0 & 0\\ -\sin\alpha_{n} & -\cos\alpha_{n} & 0 & -d_{0}\\ 0 & 0 & 1 & 0\\ 0 & 0 & 0 & 1 \end{array}]$$ (14)

$$M_{cb}=\left[\begin{array}{cccc} 1 & 0 & 0 & 0\\ 0 & \cos\beta & \sin\beta & d_{1}\sin\beta \\ 0 & -\sin\beta & \cos\beta & d_{1}\cos\beta \\ 0 & 0 & 0 & 1 \end{array}\right]$$ (15)

$$M_{ec}=[\begin{array}{cccc} 1 & 0 & 0 & -r_{1}\\ 0 & 1 & 0 & -d_{2}\\ 0 & 0 & 1 & 0\\ 0 & 0 & 0 & 1 \end{array}]$$ (16)

$$M_{de}=\left[\begin{array}{cccc} \cos\varphi_{3} & -\sin\varphi_{3} & 0 & 0\\ \sin\varphi_{3} & \cos\varphi_{3} & 0 & 0\\ 0 & 0 & 1 & 0\\ 0 & 0 & 0 & 1 \end{array}\right]$$ (17)

where *β* is the gear helix angle, *r*_*1*_ is the radius of the indexing circle of the pinion gear, and *d*_*2*_ = *φ*_3_ *r*_*1*_. ${\vec{n}}_{c}$ is the unit normal vector on the local coordinate plane; ${\vec{v}}_{c}$ is the displacement-dependent velocity vector.

**Fig 4:**
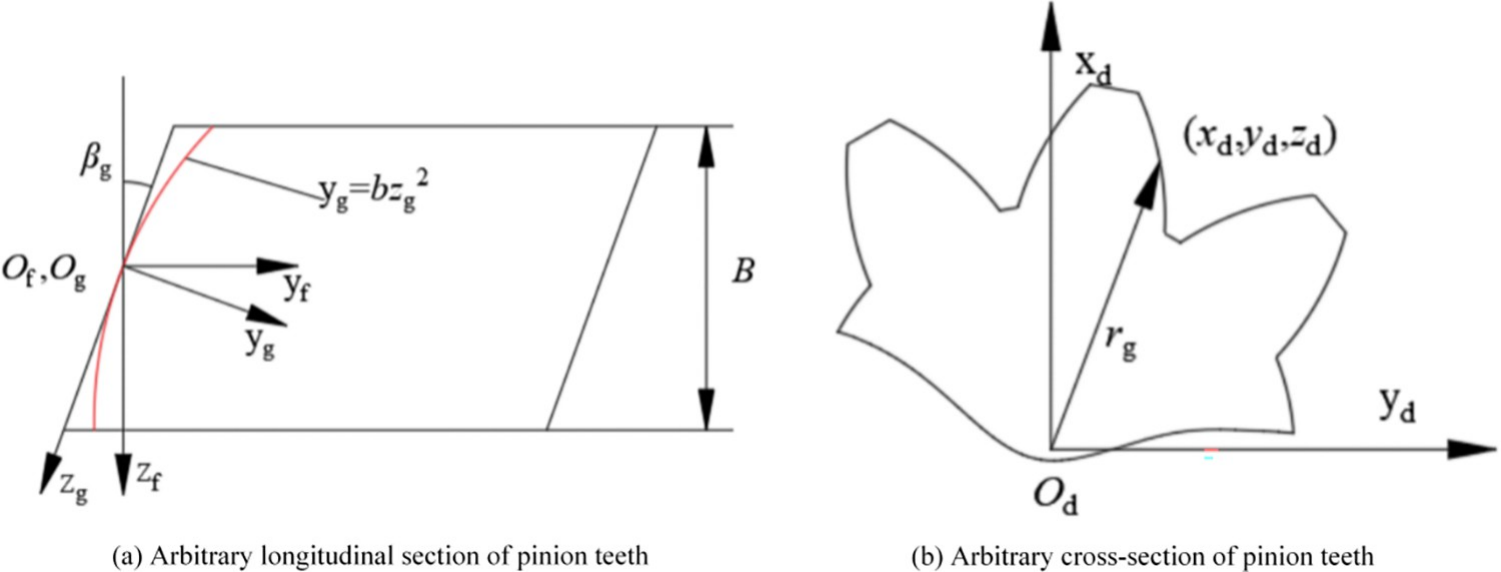
Schematic diagram of tooth profile trimming.

The parabolic profile along the helix direction is adopted for the tooth profile, and the schematic diagram of tooth profile is shown in Fig 4. The coordinate systems *S*_*f*_ and *S*_*g*_ are the auxiliary coordinate system established in the pinion tooth profile and the coordinate system along any helix angle direction, respectively. In the coordinate system *S*_*g*_:

$$y_{g}=bz_{g}^{2}$$ (18)

where, *b* is the toothwise parabolic modification factor.

In the coordinate system *S*_*f*_:

$$z_{f}=z_{g}\cos\beta_{g}+bz_{g}^{2}\sin\beta_{g}$$ (19)

$$y_{f}=-z_{g}\sin\beta_{g}+bz_{g}^{2}\cos\beta_{g}$$ (20)

The following changes will occur in the tooth coordinates after trimming:

$$\Delta y=bz_{g}^{2}\cos\beta_{g}$$ (21)

According to Eq (19) and Fig 4:

$$z_{g}=\frac{-\cos\beta_{g}+\sqrt{\cos^{2}\beta_{g}+4bz_{f}\sin\beta_{g}}}{2b\sin\beta_{g}}$$ (22)

Combine Equs (22) with (21):

$$\Delta y=b{\left(\frac{-\cos\beta_{g}+\sqrt{\cos^{2}\beta_{g}+4bz_{f}\sin\beta_{g}}}{2b\sin\beta_{g}}\right)}^{2}\cos\beta_{g}$$ (23)

among them: $\beta_{g}=\arctan(r_{g}\;\tan\beta /r_{1});r_{g}=\sqrt{x_{d}^{2}+y_{d}^{2}};z_{f}=z_{d}$

As a result, the pinion tooth equations after profile trimming and tooth orientation trimming can be obtained:

$$r_{d}(x_{a},d_{1},\varphi_{3})=M_{d}M_{ec}M_{cb}M_{ba}r_{a}(x_{a})+\left[\begin{array}{c} 0\\ \Delta y\\ 0\\ 0 \end{array}\right]$$ (24)

### 4.2 Shape modification optimization

Based on the gear teeth contact analysis conducted in this study, it is possible to modify the tooth profile within the TCA framework to achieve an optimal vibration suppression profile. To this end, this study employs a genetic algorithm to optimize both the tooth profile and the parabolic trimming coefficients along the tooth direction. The genetic algorithm is an optimization technique grounded in the principles of biological evolution, primarily employed for solving optimization challenges, including the identification of a function’s minimum or maximum values. Genetic algorithms simulate the biological evolution process by mapping the solution space of a problem onto a population of organisms. In each generation, individuals are evaluated for fitness via the fitness function, and those with higher fitness scores are selected for reproduction in the next generation, following a specific selection mechanism. Concurrently, crossover and mutation operations generate new individuals, enhancing the population’s diversity [16]. In this study, we employ genetic algorithms for the optimization of gear trimming, with the primary objective being the minimization of the gear system’s vibration acceleration root mean square (RMS) value under critical operating conditions. This approach aims to substantially reduce vibration and noise in the gear system. The process begins by establishing an initial population comprised of various randomly generated tooth profile and tooth flank parabolic trimming coefficients. The diversity within this initial population is crucial as it ensures extensive exploration of the search space. During each generation iteration, the algorithm evaluates and selects individuals based on their performance. The fitness of each individual is assessed in relation to the vibration performance of the gear system, serving as the fitness function. The optimization process involves both crossover and mutation operations. The crossover operation permits individuals to exchange segments of their genetic makeup, thereby creating novel solutions. Concurrently, the mutation operation injects new genetic variation by randomly modifying certain genes within an individual. These operations are essential for maintaining genetic diversity and avoiding premature convergence to local optima. This iterative process continues until either a predetermined number of iterations is completed or the improvement in the solution becomes negligible, falling below a specific threshold. By adhering to this methodology, our approach effectively navigates the complex optimization landscape, leading to significant reductions in vibration and noise levels within the gear system, which is pivotal for the enhancement of gear dynamics in electric vehicles. We consider the influence of working conditions on the effect of shape trimming, and take the minimum of the mean value of the root mean square of the vibration acceleration under each working condition in the whole working range as the optimization objective of the genetic algorithm, and the function expression is as follows:

$$\min\left(f\right)=\frac{\sum_{i=1}^{N}X\left(i\right)}{N}=\frac{\sum_{i=1}^{N}\sqrt{\frac{\sum_{j=1}^{M}y{\left(j\right)}^{2}}{M}}\left(i\right)}{N}$$ (25)

where, *X*(*i*) is the root-mean-square relative vibration acceleration under a certain working condition; *N* is the number of working conditions selected in the whole working range; *y* is the relative vibration acceleration in the direction of the meshing line; and *M* is the number of meshing points selected in one meshing cycle.

**Fig 5:**
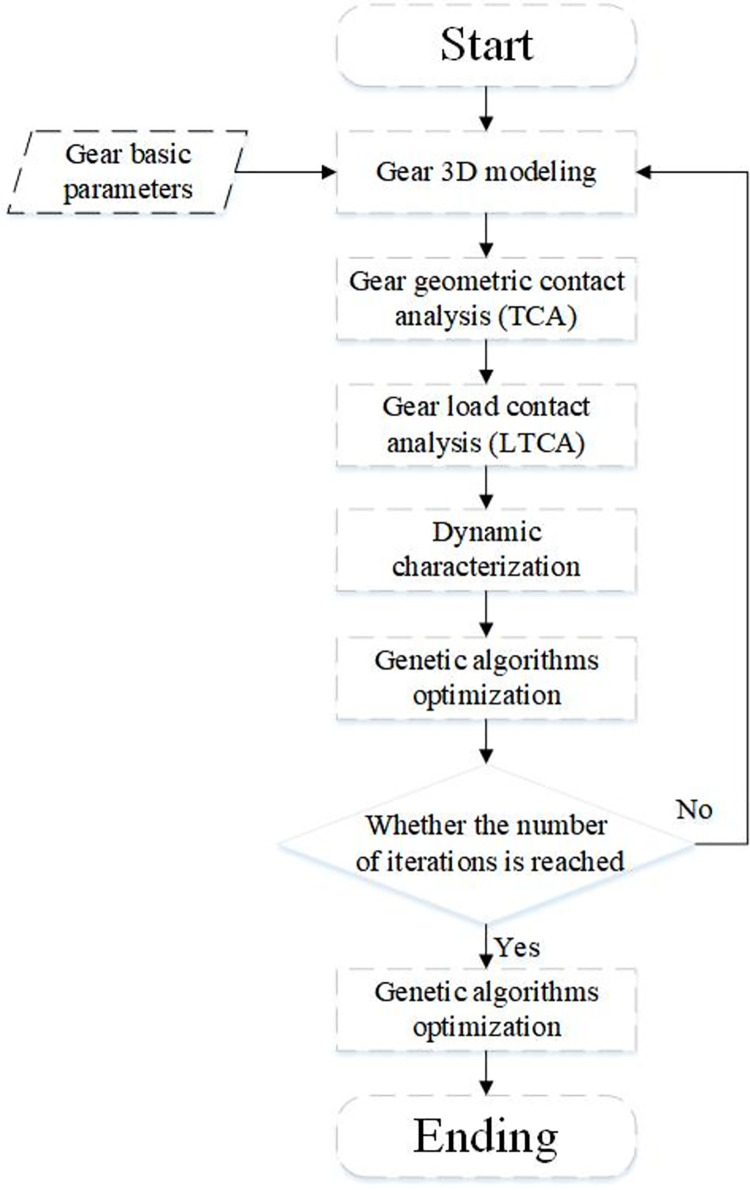
Optimization flow of trimming coefficients.

**Fig 6:**
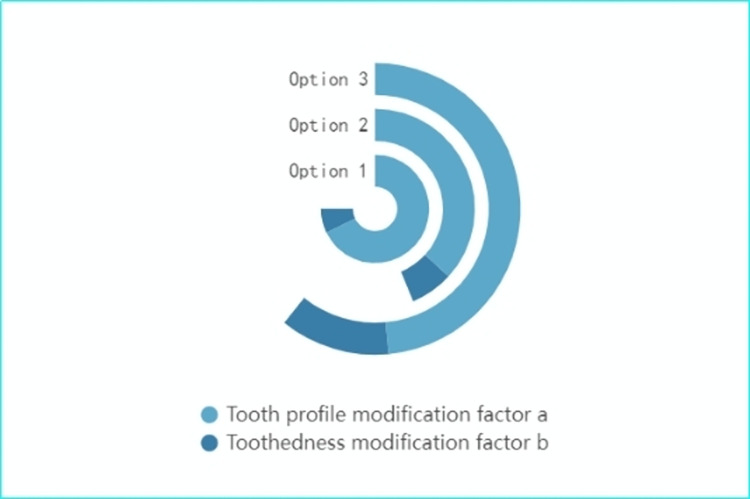
Multi-torque trimming optimization results.

The optimization process of tooth profile and tooth direction parabolic trimming coefficients based on genetic algorithm is shown in Fig 5:

In this section of our research, we compare three distinct Options, all utilizing a full speed domain trimming approach, with variations primarily in the input torque settings. Option 1: Here, the input torque is set at 85 N-m. This setting provides a baseline for evaluating the performance of the gear trimming method under a moderate torque condition. Option 2: The input torque is increased to 100 N-m in this Option. This increment allows us to assess the impact of a higher torque load on the gear system’s performance, particularly in terms of vibration and noise levels. Option 3: This Option represents the core methodology of our study. It incorporates a trimming method that integrates both the input torque and changes in rotational speed. This approach is more comprehensive, as it reflects the dynamic operational conditions of a high-speed helical gearbox drive system more accurately. For the testing of these Options, we operate the high-speed helical gearbox drive system under a preset input torque range of 70 N-m to 160 N-m, with the system running at a constant speed of 6000 rpm. This range is chosen to encompass a broad spectrum of operational conditions, from relatively low to high torque scenarios. The optimized tooth face and directional modification coefficients for each input torque setting are depicted in Fig 6.

We performed dynamic characterization of the optimized and untrimmed tooth surface under different torques, and obtained the time and frequency domain response data of the high-speed class helical gear transmission system when the input torque is 85N-m, 100N-m, 115N-m, and 130N-m.

**Fig 7:**
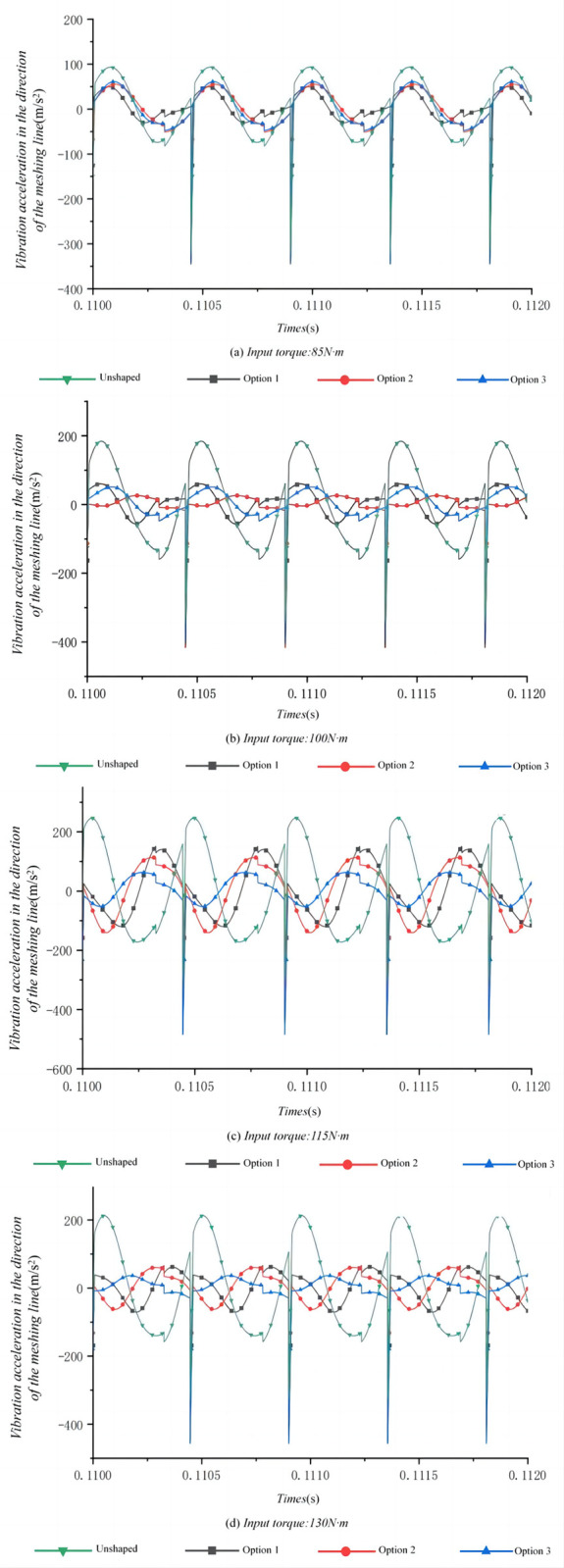
Time-domain response of high-speed stage helical gearing system under different input torque conditions.

In the study of vibrational characteristics of high-speed helical gear systems, Fig 7 demonstrates that under different input torque conditions, tooth profile modification significantly reduced the vibrational acceleration in the gear meshing line direction. From the comparative analysis of the time-domain response, at an input torque of 85 N-m, Option 1 exhibited the lowest vibrational acceleration, with a root mean square (RMS) value of 41.97 m/s^2^. This finding suggests that under lower load conditions, the tooth profile modification of Option 1 is most effective in mitigating dynamic responses. When the input torque increased to 100 N-m, Option 2 outperformed the other options, reducing the RMS value of vibrational acceleration to 40.66 m/s^2^. This result indicates that under medium load conditions, Option 2’s tooth profile modification can better balance the dynamic response of the gear system. At input torques of 115 N-m and 130 N-m, Option 3 demonstrated the best performance in reducing vibrational acceleration. This phenomenon suggests that in scenarios with higher torques, the modification approach of Option 3 might be more suitable and capable of handling higher stress levels without compromising gear meshing stability.

The effectiveness of each tooth profile modification option is closely related to the applied torque, and there is no one-size-fits-all solution. Different modification options exhibit distinct performance under varying load conditions. When considering tooth profile modifications in gear systems, it is imperative to select the appropriate option based on the system’s typical operating conditions. Choosing a specific tooth profile modification strategy based on the anticipated torque range will aid in achieving optimal results in reducing vibration and extending the lifespan of the gears.

**Fig 8:**
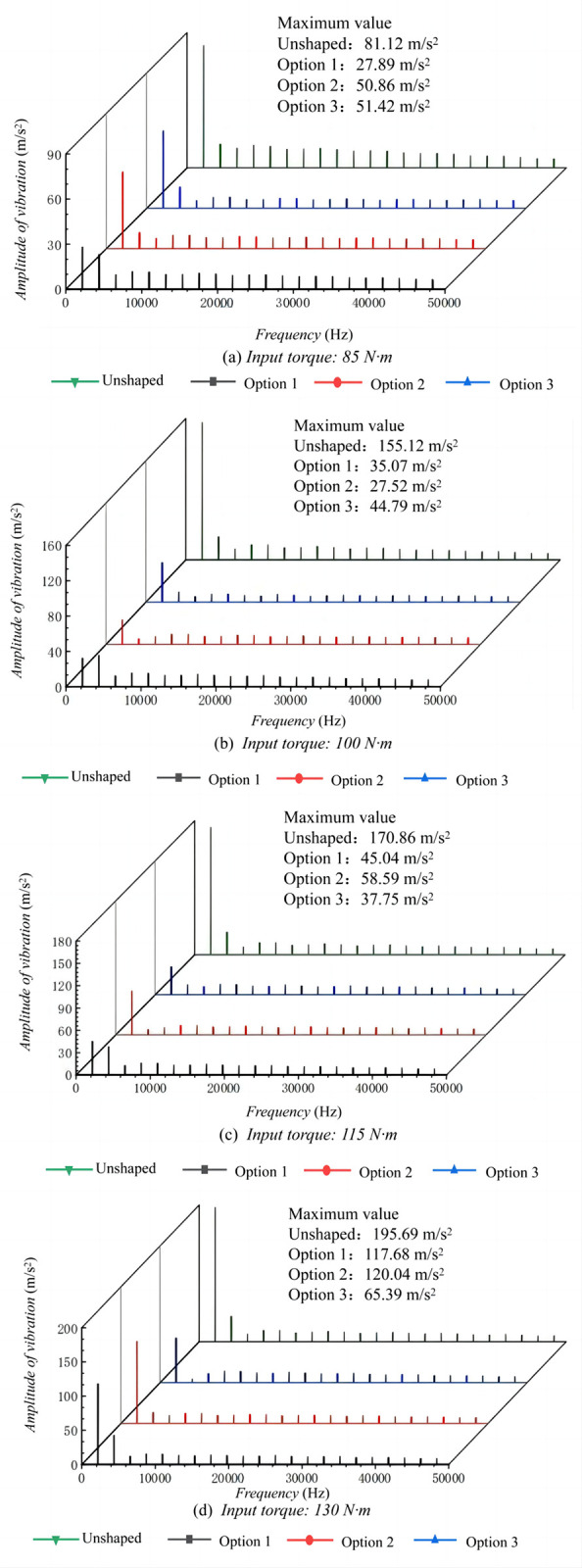
Frequency domain response of a high-speed stage helical gear drive system.

When examining the frequency domain response of high-speed gear transmission systems, Fig 8 reveals the changes in system vibrational amplitude after tooth surface modification. The peaks in the frequency analysis represent the maximum vibrational amplitude at specific frequencies, serving as crucial indicators for evaluating vibration suppression effects. As depicted in Fig 8(A), under an input torque of 85 N·m, Option 1 exhibited the minimum frequency domain vibrational amplitude, with a maximum value of 27.89 m/s^2^. At an input torque of 100 N·m, Fig 8(B) shows that Option 2 reduced the maximum vibrational amplitude to 27.52 m/s^2^, demonstrating excellent vibration suppression for both conditions. With the input torque increased to 115 N·m and 130 N·m, Option 3 showed the lowest vibrational amplitudes in Fig 8(C) and 8(D), with maximum values of 37.75 m/s^2^ and 65.39 m/s^2^, respectively. This indicates that Option 3 performs superiorly in vibration suppression under high torque conditions.

Although Options 1 and 2 exhibited the best vibration suppression effects at specific torques, namely 85 N·m and 100 N·m, Option 3 maintained relatively low vibrational levels in the gear meshing line direction across all torque conditions, even though it did not guarantee the minimum vibration at every torque level. The combined analysis of Figs 7 and 8 indicates that while Option 3 may not ensure the lowest vibration level at each torque condition, it consistently maintains low vibration in the meshing line direction across all torques, a characteristic particularly important for systems operating within a variable torque range. This underscores the significance of employing a multi-torque-point analysis in gear system design to ensure vibration control under varying operating conditions. Therefore, in designing tooth profile modification schemes, the performance of the system across the entire anticipated torque range should be considered, rather than focusing solely on a single condition. This comprehensive approach may involve the use of multi-objective optimization techniques to achieve optimal vibration control under all expected operating conditions.

**Fig 9:**
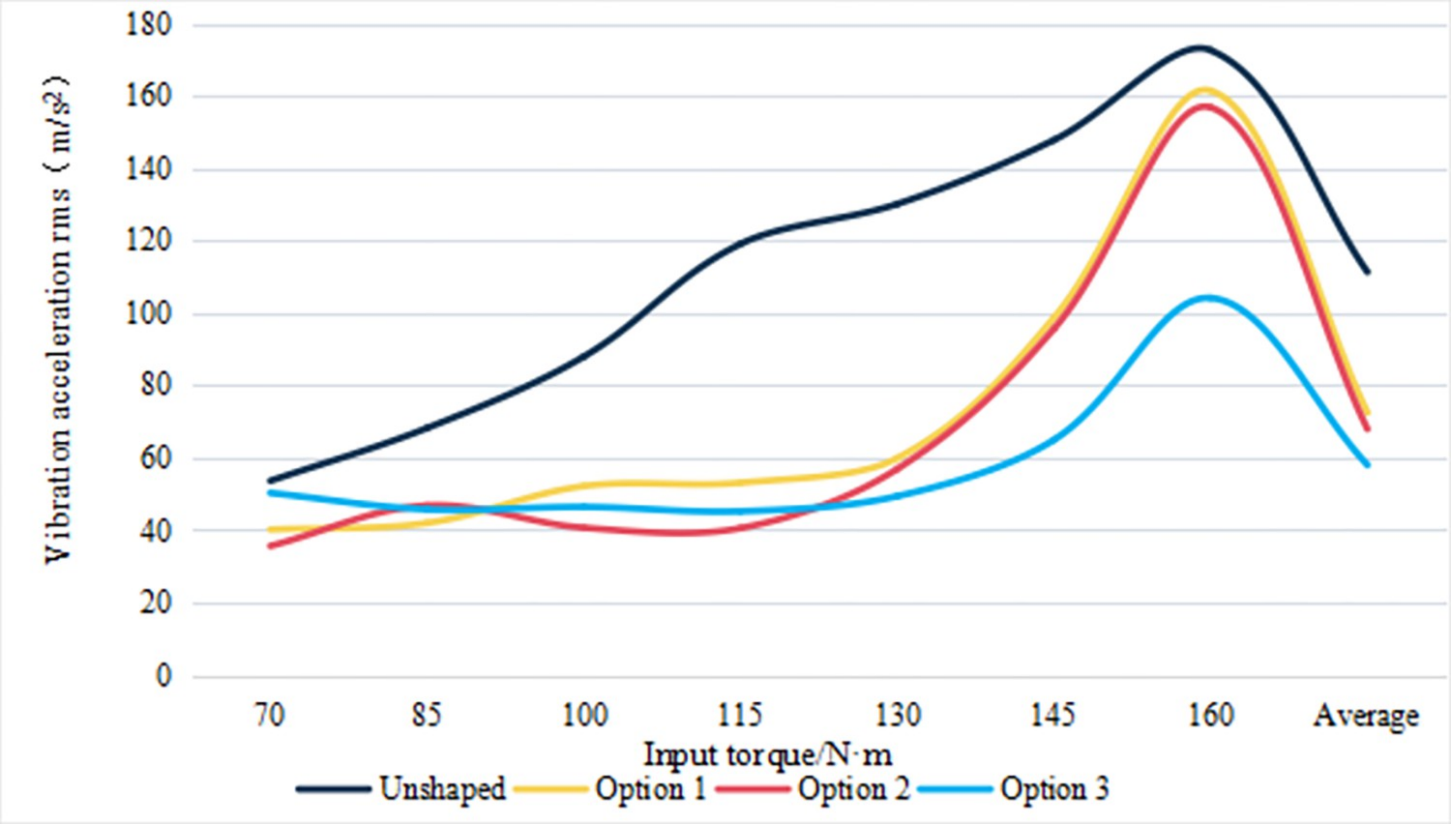
Root-mean-square of vibration acceleration in the direction of the meshing line for different input torques (m/s^2^).

For a more visual comparison of the vibration performance between the different Options, the results of the root mean square of the vibration acceleration of the high-speed stage helical gear drive system at input torques of 70N-m, 85N-m, 100N-m, 115N-m, 130N-m, 145N-m and 160N-m are shown in Fig 9.

In Fig 9, the root-mean-square (RMS) values of vibration acceleration for different gear profiles are scrutinized under variable input torques. The unshaped profile, serving as a baseline, demonstrates an increasing trend in RMS vibration acceleration with the highest recorded value at 172.6 m/s^2^ and an average of 111.35 m/s^2^ across the torque spectrum. Option 1 optimizes vibration suppression at an 85 N-m input torque, attaining a notable RMS reduction to 41.97 m/s^2^, with an average decrease to 72.47 m/s^2^. Option 2 presents its lowest vibration accelerations at 35.62 m/s^2^ for 70 N-m, 40.66 m/s^2^ for 100 N-m, and 40.55 m/s^2^ for 115 N-m input torques, achieving an average RMS of 67.95 m/s^2^. Option 3 exhibits a consistent vibration suppression capability, particularly beyond 130 N-m, where it records a lower RMS of 45.2 m/s^2^, culminating in the lowest average RMS value of 58.02 m/s^2^ among all options. The comparison indicates that while Options 1 and 2 offer significant vibration reductions at specific torque intervals, Option 3 provides a more uniform suppression across the entire torque range. The data suggests that Option 3’s design may be preferable for applications that encounter a wide range of torques and demand consistent vibration mitigation. This analysis underscores the critical role of gear profile modifications in enhancing mechanical system performance by reducing vibrational disturbances, with implications for the durability and operational smoothness of gear assemblies.

### 4.3 Optimal trimming in the full-speed domain

**Fig 10:**
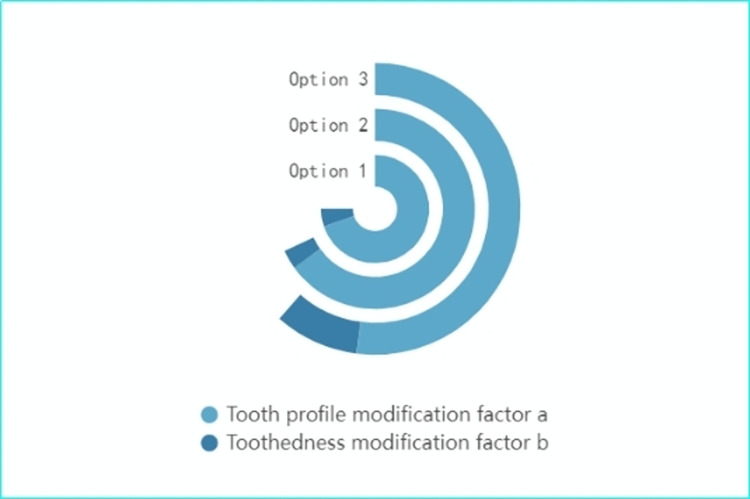
Optimization results of full speed domain shape trimming.

In this section of the study, Options 1 and 2 both employ the full torque trimming method; however, they differ in terms of input speed. Option 1 is configured with an input speed of 5000 rpm, while Option 2 operates at a higher input speed of 7000 rpm. Option 3, on the other hand, represents the approach central to this study. It employs a trimming method that integrates input torque with variations in speed. This methodology embodies a more comprehensive approach, considering both torque and speed changes, thus offering a more nuanced understanding and optimization of gear performance. Under the defined operating conditions for the high-speed stage helical gear transmission system, with an input torque of 80 N-m and an operating speed range extending to 15,000 rpm, this study adopts the gear shaping method outlined in the preceding section and utilizes a genetic algorithm to optimize the shaping of the tooth flanks for designated input speeds. Concurrently, to fulfill the full-speed domain requirements, input speeds of n = 3000rpm, 4000rpm, 5000rpm, 7000rpm, 8000rpm, and 12000rpm, among other discrete operating conditions, were chosen to optimize the tooth profile. The outcomes of the optimization, including the tooth profile and the parabolic trimming coefficients in the tooth direction, are illustrated in Fig 10.

Experiments were carried out to examine the dynamic characteristics of both trimmed and untrimmed tooth flanks, tested across varying speeds. This aimed to obtain the time and frequency domain responses of a high-speed class helical gear transmission system at input speeds of 2000 rpm, 4000 rpm, 6000 rpm, and 8000 rpm. The findings of the experimental analysis indicate that employing a trimmed tooth surface markedly enhances the system’s dynamic performance and effectively mitigates vibration and noise.

**Fig 11:**
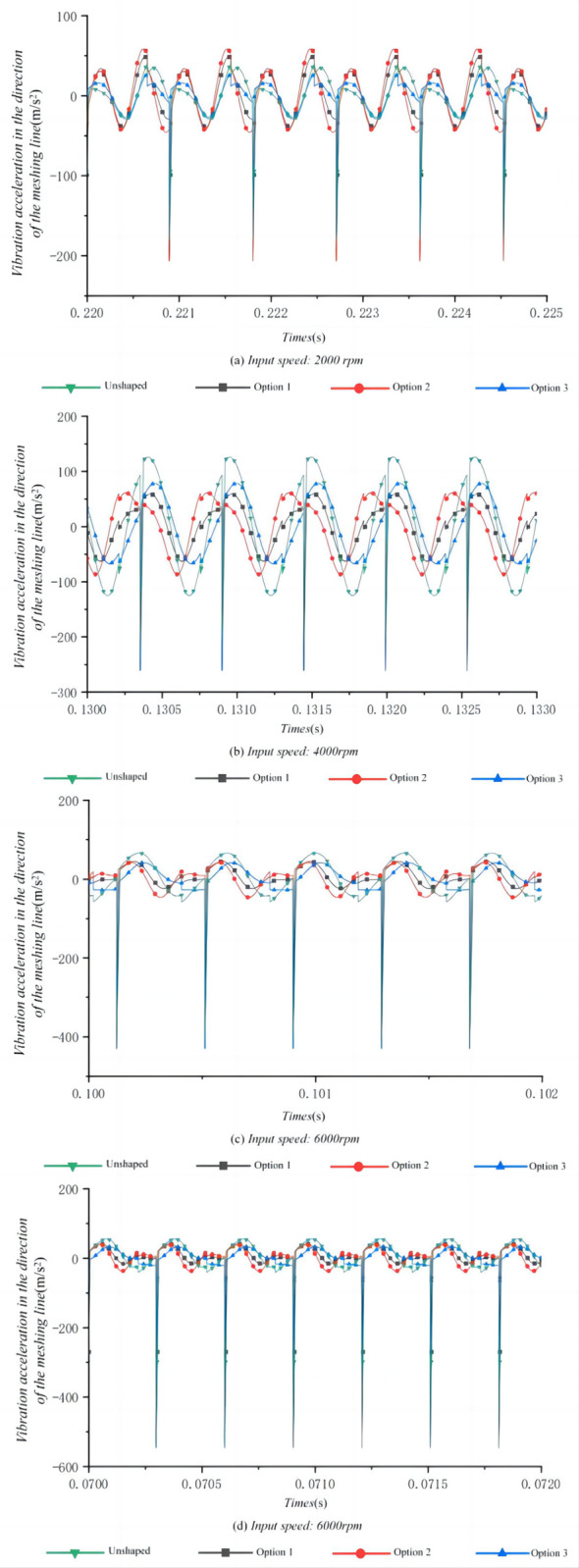
Time domain response of high speed stage helical gear train at different input speeds.

As shown in Fig 11,option 1 exhibits a significant vibration suppression effect at 2000 rpm with a maximum amplitude of 38.96 m/s^2^. At 4000 rpm, it continues to show a relatively effective vibration control with the amplitude peaking at 42.91 m/s^2^. However, there is an improvement at higher speeds with lower maximum amplitudes of 27.72 m/s^2^ and 30.84 m/s^2^ at 6000 rpm and 8000 rpm respectively. Exhibiting the highest maximum amplitude at 4000 rpm with 60.57 m/s^2^, Option 2 suggests less effectiveness in vibration suppression at this speed. However, similar to Option 1, it shows improved performance at higher speeds with 27.48 m/s^2^ and 28.43 m/s^2^ at 6000 rpm and 8000 rpm respectively. Option 3 shows the lowest maximum amplitude at 2000 rpm with 18.34 m/s^2^, indicating the best vibration suppression among the options at this speed. Although it shows the highest amplitude at 4000 rpm with 70.41 m/s^2^, it performs better at 6000 rpm and 8000 rpm with lower maximum amplitudes. The unshaped gear consistently shows higher maximum amplitudes compared to the reshaped options, particularly at higher speeds. At 4000 rpm, the amplitude reaches 113.88 m/s^2^, which is substantially higher than any of the reshaped options. This trend continues at 6000 rpm and 8000 rpm with the unshaped gear exhibiting the highest amplitudes.

These results suggest that the reshaping options can effectively reduce the vibration amplitudes compared to the unshaped gear, particularly at higher operating speeds. Option 3 demonstrates an overall balanced performance across the entire speed range, despite not always having the lowest amplitude at individual speeds. The improved performance of the reshaped gears at higher speeds indicates that reshaping is more beneficial under conditions that are more demanding, which could be due to higher speeds generating more significant dynamic effects that the reshaping can mitigate.

**Fig 12:**
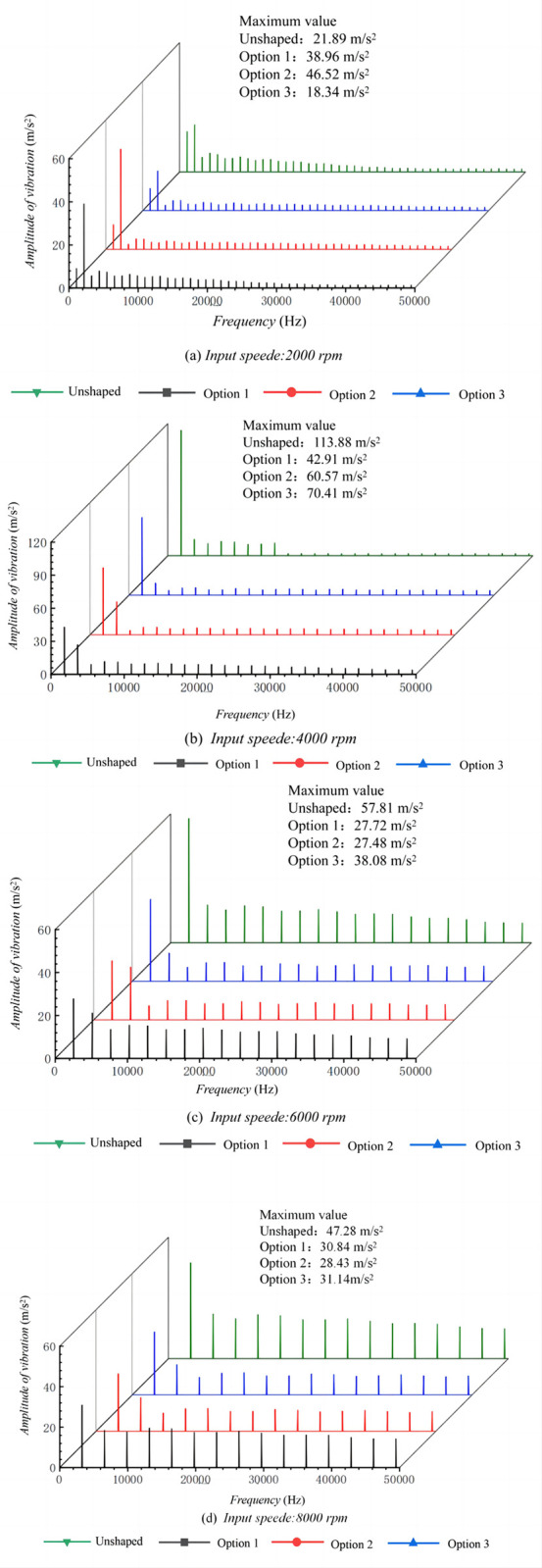
Frequency domain response of high speed stage helical gear train at different input speeds.

Fig 12 depicts the frequency domain response of the high-speed helical gear system. At an input speed of 2000 rpm, the frequency domain vibration amplitudes for Options 1 and 2 exceed those of the unmodified tooth surface. However, at higher speeds, modified tooth surfaces significantly reduce the frequency domain vibration amplitudes. Specifically, at an input speed of 4000 rpm, Option 1 exhibits the smallest frequency domain vibration amplitude, with a peak value of 42.91 m/s^2^, indicating optimal shaping effects at this specific rotational speed. At input speeds of 6000 rpm and 8000 rpm, Option 2 demonstrates the smallest frequency domain vibration amplitudes, with peak values of 27.48 m/s^2^ and 28.43 m/s^2^, respectively, suggesting superior vibration damping at these higher speeds. Option 3, considering full-speed domain shaping, significantly reduces the frequency domain vibration amplitude only at 2000 rpm. Although the frequency domain vibration minimizes at 2000 rpm, Option 3 consistently lowers the frequency domain vibration amplitude across all speeds compared to the unmodified option.

These observations indicate that tooth surface modification effectively reduces vibration amplitudes across the entire operational speed range, particularly at higher operational speeds. While a single option does not always yield the lowest vibration amplitude at specific speed points, considering full-speed domain shaping effects provides better vibration damping performance across various speeds. This analysis underscores the importance of selecting the optimal shaping strategy based on the operational speed range of the gear system, which is crucial for gear design in variable speed applications.

**Fig 13:**
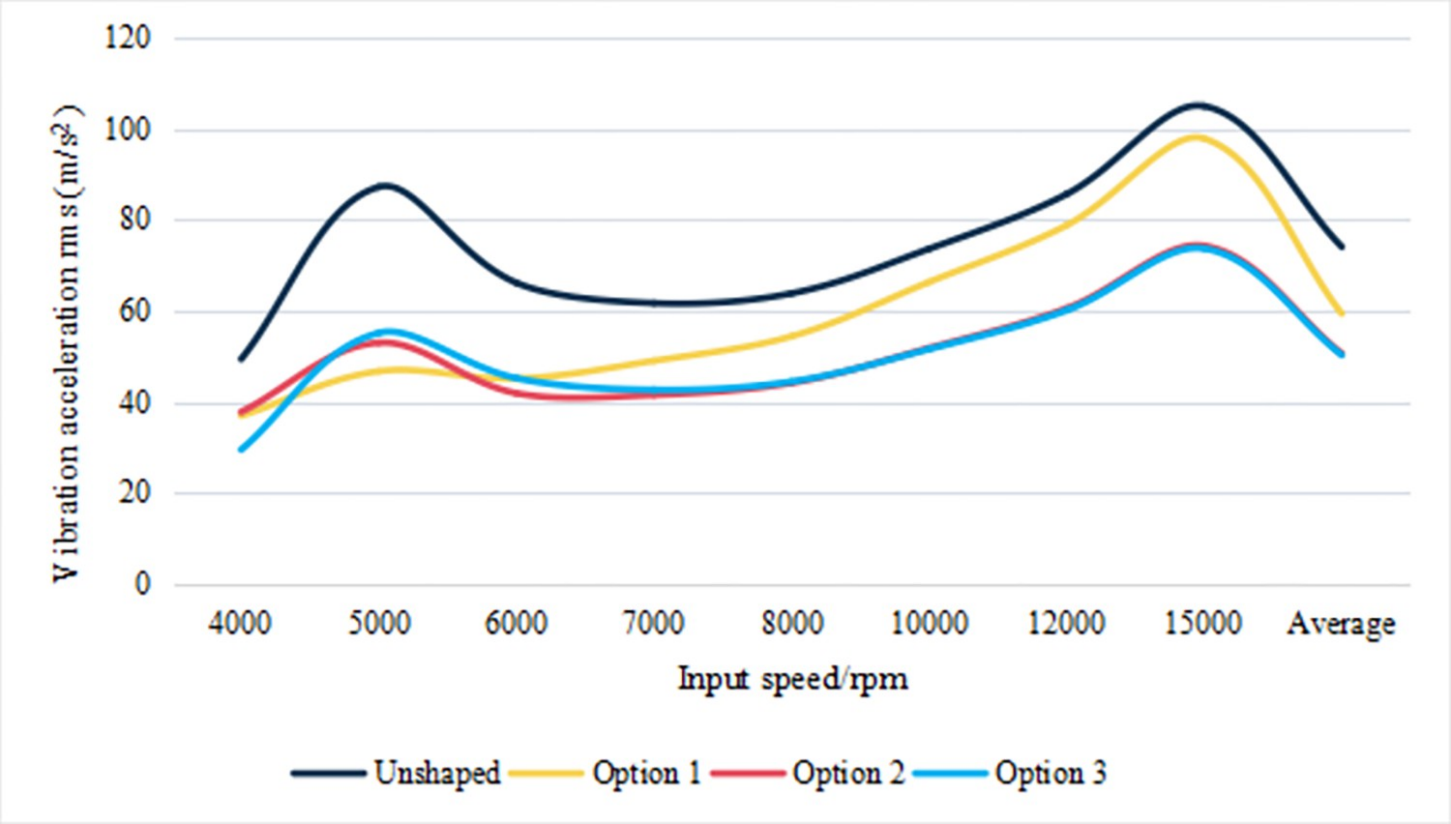
Root mean square of vibration acceleration in the direction of the meshing line at different rotational speeds (m/s) ^2^.

Fig 13 illustrates the root mean square (RMS) values of vibration acceleration along the meshing line for unshaped and shaped gear profiles under variable rotational speeds. The analysis of these values underlines the effectiveness of gear modifications in vibration suppression. At 5000 rpm, Scheme 1 achieves a significant reduction in tooth acceleration RMS, registering at 46.77 m/s^2^, which is a 46.40% decrease from the unshaped profile. This pronounced reduction highlights Scheme 1’s capacity for substantial vibration mitigation at this operational speed. Conversely, Scheme 2 proves most efficacious at intermediate speeds, specifically at 6000, 7000, and 8000 rpm, with recorded RMS accelerations of 42.04 m/s^2^, 41.65 m/s^2^, and 44.30 m/s^2^, respectively. Such findings suggest Scheme 2’s optimized performance in the targeted speed domain, affording it an advantageous position for applications operating predominantly within this range. Scheme 3, encompassing full-speed domain trimming, does not uniformly deliver the lowest RMS values at discrete speed intervals but exhibits a consistent reduction in vibration levels throughout the spectrum. Notably, at speeds of 4000 rpm and surpassing 10000 rpm, Scheme 3 demonstrates efficacious vibration suppression. The scheme’s average RMS of 50.3325 m/s^2^, the lowest among the profiles analyzed, confirms its comprehensive effectiveness over the entire speed range. Collectively, the data delineated in Fig 13 convey that strategic gear profile modifications are pivotal in attenuating vibrational impacts across diverse operational speeds. In particular, Scheme 3’s design optimization is indicative of its potential to provide the most uniform vibration reduction, serving as an integral consideration for enhancing gear system reliability and performance in engineering applications.

**Fig 14:**
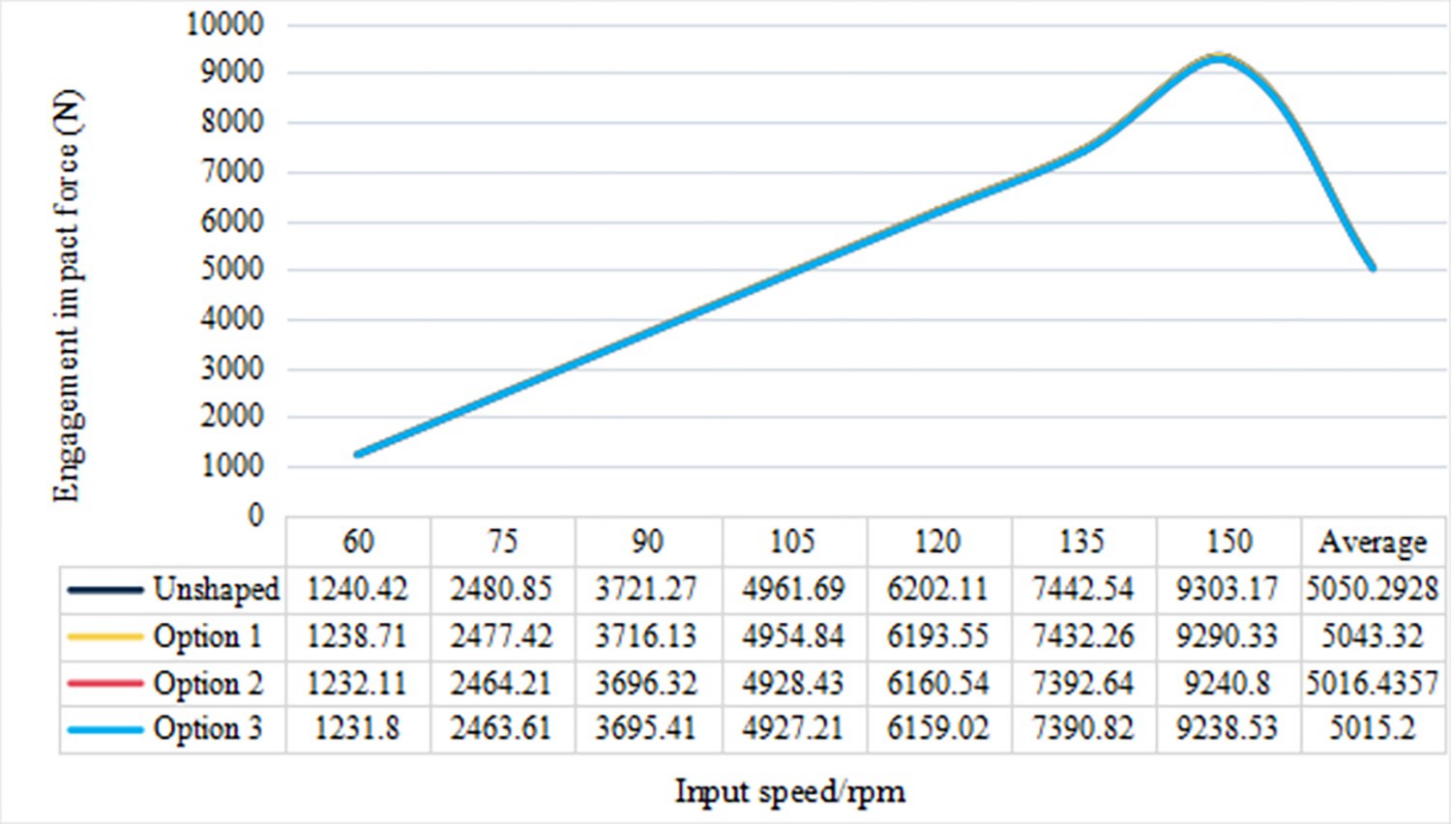
Amplitude of engagement impact force.

In Fig 14, the amplitude of engagement impact force across different gear profile modifications is quantitatively depicted, offering a compelling comparison of the impact forces at varying input speeds. The unshaped gear profile exhibits the highest average impact force of 5050.29 N, setting a baseline for the effectiveness of subsequent modifications. Options 1 and 2 demonstrate a marginal reduction in the average impact force to 5043.32 N and 5016.44 N, respectively, indicating a moderate enhancement in gear engagement. Option 3, however, markedly outperforms the aforementioned configurations by reducing the average force to 5015.20 N, underscoring a substantial improvement in minimizing engagement impact force. Further scrutiny reveals a pivotal reduction in the peak impact forces for the modified profiles, particularly at high input speeds. While the unshaped profile peaks at 9303.17 N at 135 rpm, Option 3 attenuates this peak by approximately 365.17 N at 150 rpm, signifying a superior capacity in managing impact forces during high-speed operations. Additionally, although all profiles display a proportional increase in impact force with input speed, the modified profiles, especially Option 2, exhibit a tempered escalation at higher velocities, hinting at an enhanced resistance to vibration-induced forces. Collectively, the data elucidate that gear profile modifications yield a significant decrement in engagement impact forces, with pronounced efficacy at elevated speeds. Among the evaluated modifications, Option 3 emerges as the most effective, likely due to an optimized design that mitigates system vibration and enhances operational smoothness.

## 5. Discussion

This paper presents an optimized gear forming method for electric vehicles, taking into account variations in input torque and speed. It aims to address vibration and noise issues in gear trains, which arise from dynamic excitation, elastic deformation, and errors in gear manufacturing and installation. Previous studies have concentrated on error measurement and accuracy but have not provided an integrated gear-forming method that accounts for various factors. This study introduces an optimized gear forming technique, enhancing gear reliability and mitigating noise and vibration at high speeds. It employs techniques such as tooth contact analysis (TCA) and loaded tooth contact analysis (LTCA), alongside various mathematical and experimental methods, to analyze how variations in input torque and speed affect gear vibration.

Compared to existing studies, this method exhibits superior vibration suppression across multiple torque levels and the full-speed domain. Notably, this method surpasses traditional shaping strategies in full-speed domain shaping under single operating conditions. While existing research has explored gear reshaping’s impact on vibration, this study achieves improved vibration control under various operating conditions through optimization via genetic algorithms [17–21]. This study not only theoretically introduces a novel gear trimming method but also provides experimental verification of its effectiveness. This holds significant practical implications for designing and optimizing high-speed gears in electric vehicles, particularly for noise reduction and reliability enhancement. Vibration suppression can be achieved through tooth profile modification, thereby improving gear operation stability in electric vehicles by considering the effects of torque and speed [22–28]. Utilizing TCA and LTCA analysis techniques, this method achieves optimization of the gear tooth profile and its coefficients. This allows for an analysis of transmission characteristics under various excitations, optimizing and enhancing the current profile solution. It also enhances the profile technology’s applicability to electric vehicles across diverse working conditions [29–32].

Despite the study’s positive outcomes, it has limitations, including the incomplete consideration of the gear system’s working time distribution under various operating conditions. Future research could focus on integrating weighting factors for the working time distribution to more comprehensively optimize the tooth profile shaping strategy. Additionally, future studies should account for motor efficiency’s impact on the gear system’s input torque and speed for a more precise and comprehensive optimization of gear shaping.

## 6. Conclusions and outlook

### 6.1 Conclusion

The high-speed class helical gears in this study are optimized using Tooth Contact Analysis (TCA) and Loaded Tooth Contact Analysis (LTCA), with genetic algorithms employed to fine-tune the tooth profile and parabolic coefficients. This approach enables us to achieve the most effective trimmed tooth profiles under multi-torque and full-speed domain conditions. Comparative analysis of the profile trimming schemes under specific loads and speeds revealed that the optimized gears exhibit superior vibration control in experimental tests. Notably, the present method achieves a 47.89% reduction in vibration compared to untrimmed gears under varying input torques, outperforming traditional single-condition trimming methods. Similarly, under different input speeds, our method registers a 32.08% reduction in vibration compared to untrimmed gears, again surpassing single-condition methods. The overall vibration amplitude under both conditions is more stable and maintained at a lower level, demonstrating the effectiveness of our optimization in enhancing the dynamic stability of the gear system. Consequently, the Electric Vehicle (EV) gear reshaping method, which considers both input torque and speed, shows significantly better reshaping effects and global adaptability than methods focusing on a single working condition.

### 6.2 Outlook

While the current study utilizes the average value of the root mean square (RMS) of vibration acceleration across multiple conditions as a metric for shape modification merit, it overlooks the variation in working time for each condition of the reducer. Future research could address this gap. To more accurately mirror actual engineering scenarios, future studies could introduce weighting coefficients based on the proportional duration of each working condition within the operational process. Moreover, although the selection of working conditions is based on the motor’s external characteristic curve, motor efficiency has not been comprehensively accounted for. The efficiency of the motor influences the actual input torque and speed of the gear system; thus, future studies should also account for the variations in working conditions prompted by motor efficiency to more thoroughly optimize the tooth profile shaping strategy.
